# On the occurrence of the fireworm *Eurythoe complanata* complex (Annelida, Amphinomidae) in the Mediterranean Sea with an updated revision of the alien Mediterranean amphinomids

**DOI:** 10.3897/zookeys.337.5811

**Published:** 2013-09-30

**Authors:** Andrés Arias, Rômulo Barroso, Nuria Anadón, Paulo C. Paiva

**Affiliations:** 1 Departamento de Biología de Organismos y Sistemas (Zoología), Universidad de Oviedo, Oviedo 33071, Spain; 2 Pontifícia Universidade Católica do Rio de Janeiro, Rio de Janeiro, Brazil; 3 Museu de Zoologia da Unicamp, Campinas, SP, Brazil; 4 Departamento de Zoologia, Instituto de Biologia, Universidade Federal do Rio de Janeiro (UFRJ), Rio de Janeiro, RJ, Brasil

**Keywords:** Alien polychaetes, cryptic species, Gibraltar Strait, Lessepsian migrant

## Abstract

The presence of two species within the *Eurythoe complanata* complex in the Mediterranean Sea is reported, as well as their geographical distributions. One species, *Eurythoe laevisetis*, occurs in the eastern and central Mediterranean, likely constituting the first historical introduction to the Mediterranean Sea and the other, *Eurythoe complanata*, in both eastern and Levantine basins. Brief notes on their taxonomy are also provided and their potential pathways for introduction to the Mediterranean are discussed. A simplified key to the Mediterranean amphinomid genera and species of *Eurythoe* and *Linopherus* is presented plus an updated revision of the alien amphinomid species reported previously from the Mediterranean Sea. A total of five exotic species have been included; information on their location, habitat, date of introduction and other relevant features is also provided.

## Introduction

Introductions of alien species are threatening the economic and ecological well-being of marine ecosystems worldwide. The impacts of alien species on their new environments include alterations of established food webs, importation of new diseases or parasites, competition with native species for food and space, and even changing gene pools (Occhipinti-Ambrogi et al. 2011; Cosentino and Giacobbe 2011; Arias et al. 2013a, 2013b; Çinar 2013). Invaders are able to modify the receiving ecosystems, re-structuring the ecological relations within communities, altering evolutionary processes and causing dramatic changes in native populations. Over 80% of alien polychaete species recorded to date in the Mediterranean Sea come from the Red Sea and the Indo-Pacific (Çinar 2013), presumably reaching the Mediterranean through the Suez Canal and being considered as Lesseptian migrants or Erythrean species (Por 1978). The remaining ~20% originate from the Atlantic Ocean and were introduced to this region mainly via “shipping” (Çinar 2013). In recent decades, the rate of polychaete invasions has exponentially increased and currently in the Mediterranean Sea the number of alien polychaete species is roughly 100 (Zenetos et al. 2012; Çinar 2013).

Amphinomidae is a well-known family of polychaetes that is globally distributed, reaching its highest diversity in shallow tropical and subtropical waters (Kudenov 1995) and occurring at all depths, including abyssal areas (Kudenov 1993). Large tropical species of amphinomids are normally colourful and commonly referred as “fireworms” with hollow calcareous harpoon-type chaetae containing complanine, a trimethylamine compound that cause intense irritation on skin after penetrating the skin of anyone handling them roughly (Kudenov 1993, 1995; Nakamura et al. 2008). The parapodia are biramous with dense bundles of chaetae. The notopodium bears a single true dorsal cirrus (lateral cirrus) and some species may have a second accessory dorsal cirrus (branchial cirrus). The neuropodium has a single ventral cirrus. Besides having calcareous instead of chitinous chaetae, as present in other polychaetes, most amphinomids and other members of the Amphinomida have well-developed nuchal organs known as caruncles, which extend back mid-dorsally for several segments (Kudenov 1995, Rouse and Pleijel 2001).

Shallow water forms play an important ecological role mainly in rocky and coral reef environments, where species such as *Hermodice carunculata* (Pallas, 1766) are major predators of both soft corals (Alcyonacea) and hard corals (Scleractinia) (Ott and Lewis 1972, Vreeland and Lasker 1989). Furthermore, *Hermodice caranculata* is known to act as reservoir and vector of pathogens associated with coral bleaching (Sussman et al. 2003). Another common shallow-water species is *Eurythoe complanata* (Pallas, 1766), which has been traditionally considered as having a wide circumtropical distribution. Nevertheless, recently it was demonstrated that *Eurythoe complanata* is actually a species complex. The phylogeographic analysis performed by Barroso et al. (2010) identified three closely related species forming a species complex: two species (one from eastern Pacific and the other from the Atlantic) are morphologically identical and fit the description of *Eurythoe complanata*; and the third one, slightly morphologically different from the others, corresponds to the species *Eurythoe laevisetis*. Thereby, we are here proposing the term ‘morphospecies’ to refer to *Eurythoe complanata* and *Eurythoe laevisetis*, a concept that will be explored in the discussion below. Recently *Eurythoe* cf. *complanata* was also reported from the eastern and central Mediterranean (Barroso et al. 2010, Arias et al. 2013a respectively) but its presence in the Mediterranean Sea was questioned (Zenetos et al. 2010, 2012). Therefore, in order to elucidate the current status of this species complex in the Mediterranean and update its taxonomy, specimens previously identified as *Eurythoe complanata* collected from the central and eastern Mediterranean were morphologically re-examined, taking into account the new data for this species complex. Additionally, an updated key to currently known genera and five alien species in Mediterranean Amphinomidae is included.

## Methods

**Figure 1.**
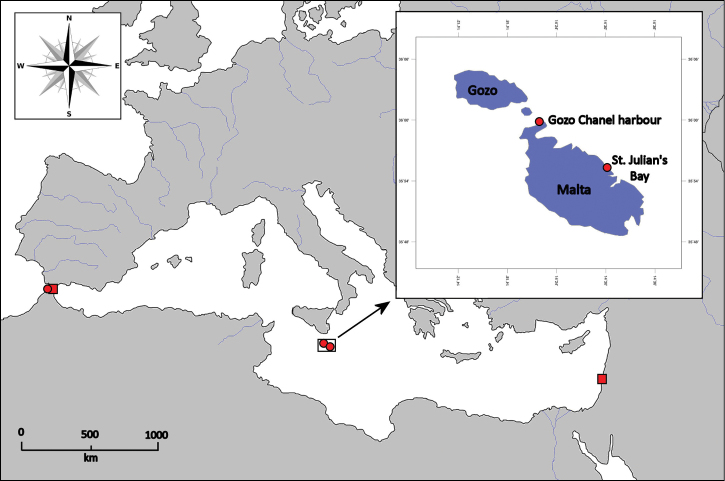
Current distribution of *Eurythoe laevisetis* (red circles) and *Eurythoe complanata* (red squares) along the Mediterranean Sea.

Field collections were made along the Maltese Islands, Central Mediterranean, on hard substrata from the shallow subtidal rocky areas at Ċirkewwa Harbour (35°59'N, 14°19'E) and St. Julian’s Bay (35°55'N, 14°29'E) in March 2011 (Figure 1). Large specimens were randomly removed by a swift hand motion. Small specimens were collected using grabs and screened using a 1 mm mesh sieve. The worms were removed from the residue under a stereomicroscope. Then, all specimens were relaxed in MgCl_2_ isotonic with seawater, fixed in 10% formaldehyde solution, rinsed in fresh water and finally transferred to 70% ethanol. Photographs were taken using a stereomicroscope Nikon SMZ-1000 equipped with a digital camera; before photography, specimens were stained with lithic carmine solution. Lithic carmine staining increased the contrast of some morphological structures, such as caruncle, branchiae, parapodial lobes and cirri. Glycerol slides of parapodial sections, examined under a compound light microscope Leica DM 2500, were used for the detailed examination of chaetal morphology and distribution.

The examined material was deposited at the Invertebrate Collection of the Department of Biology of Organisms and Systems (BOS) of University of Oviedo. Detailed location data is given below in the ‘Material examined’ sections of the respective species. The number of specimens in each sample is given in parentheses after the museum abbreviation and registration number. Furthermore, preserved specimens identified as *Eurythoe complanata* from the Gibraltar Strait, eastern Mediterranean (deposited in the MNCN), and the coasts of Atlit, Israel (deposited in the BMNH), were re-examined.

Additionally, comparative material was also studied: *Eurythoe laevisetis* Fauvel, 1914: São Tomé Island: IBUFRJ 0545; *Eurythoe* cf. *laevisetis*: Sal Island (Cape Verde): BOS-Amp1; Gran Canary (Canary Islands): BOS-Amp2; *Eurythoe complanata*: Bocas del Toro, Panamá (Caribbean): IBUFRJ 0542. Red Sea (unknown locality): BMNH 1923.3.20.8.

### Abbreviations

BMNH The Natural History Museum, London, U.K.

BOS Biology of Organisms and Systems, University of Oviedo, Spain

IBUFRJ Instituto de Biologia, Universidade Federal do Rio de Janeiro, Brazil

MNCN Museo Nacional de Ciencias Naturales, Madrid, Spain

An updated check-list of the alien amphinomid species is provided based on an exhaustive review of the species records in the literature. The species data were mainly extracted from the regional reviews on alien species and compilations of polychaete species. We have also included data on their ecology, distribution and other relevant features.

## Results

The revision of the literature along with our results (observations on 28 Mediterranean specimens belonging to *Eurythoe complanata* complex) revealed that five amphinomid species belonging to three genera were determined to be alien species in the Mediterranean Sea: *Eurythoe laevisetis*, *Eurythoe complanta*, *Linopherus acarunculatus* (Monro, 1937), *Linopherus canariensis* Langerhans, 1881 and *Notopygos crinita* Grube, 1855. The diagnostic differences between these species are summarised in the key provided. Furthermore, information about location, habitat, date of introduction and other relevant features are provided in Table 1.

**Table 1.** Summary of current knowledge on exotic Mediterranean Amphinomidae.<br/>

**Species**	**Locality**	**Year**	**Mediterranean area**	**Habitat**	**Others features**	**Reference**
*Eurythoe complanata* (Pallas, 1766)	Atlit (Israel)	1937	Eastern			Monro 1937, Current work
	Gulf of Eilat (Israel)	1976	Eastern	intertidal reefs of *Dendropoma* spp	Occurring together with another amphinomid *Linopherus acarunculatus*	Ben-Eliahu 1976
	Isabel II Island Gibraltar Strait (Spain)	September 1992	Western	On rocks, 3–6 m depth	Occurring sympatrically with *Eurythoe laevisetis*	Current work
	Isabel II Island Gibraltar Strait (Spain)	July 1993	Western	On rocks, 3–6 m depth		
	Congreso Island Gibraltar Strait (Spain)	July 1993	Western	On rocks, 3 m depth		Current work
	Chafarinas Islands Gibraltar Strait (Spain)	1995	Western	Rocky substrate		López 1995
*Eurythoe laevisetis* Fauvel, 1914	Isabel II Island Gibraltar Strait (Spain)	September 1992	Western	On rocks, 3–6 m depth	Occurring sympatrically with *Eurythoe complanata*	Current work
	Isabel II Island Gibraltar Strait (Spain)	July 1993	Western	On rocks, 3–6 m depth		
	Gozo Harbour (Malta)	March 2011	Central	Rocky bottom 0.5–1 m depth	Associated with the invasive *Branchiomma bairdi*	Current work
*Linopherus acarunculatus* (Monro, 1937)	Lebanon	1966	Eastern	Shallow waters	Referred to as *Pseudeurythoe acarunculata* Monro, 1937. Çinar (2009) suggest that these records could be *Linopherus canariensis* Langerhans, 1881	Laubier 1966
	Gulf of Elat (Israel)	1976	Eastern	Intertidal reefs of *Dendropoma* spp		Ben-Eliahu 1976
*Linopherus canariensis* Langerhans, 1881	Kemer (Turkey)	July, 1993	Eastern	5 m depth on algae		Çinar 2009
	Cyprus	May 1997	Eastern	35 m depth on sandy substrate	Associated with *Brachiomma lanceolatum*	
	Antalya Bay (Turkey)	1997	Eastern		Referred to as *Pseudeurythoe acarunculata* Monro, 1937	Ergen and Çinar 1997
	Cyprus	2005	Eastern			Çinar 2005
	Turkey	September-October 2005	Eastern	On rocks between 0.1–5 m. Mainly in *Calendula officinalis* substrate		Çinar 2009
	Italy	2005	Central			Occhipinti-Ambrogi et al. 2011
	Lake of Faro (Italy)	May 2008	Central	Artificial modules with a neighboring sandy bottom, 1.2 m depth	Showed an invasive behaviour, reaching densities of 41.86 ind / m^2^	Cosentino and Giacobbe 2011
*Notopygos crinita* Grube, 1855	Italy	1983	Central		Currently this species is considered as not established in the Mediterranean (Zenetos et al. 2010; Occhipinti-Ambrogi et al. 2011)	Zenetos et al. 2010

### Family Amphinomidae Lamarck, 1818
Genus *Eurythoe* Kinberg, 1857

**Type species.** *Eurythoe capensis* Kinberg, 1857, subsequent designation: *Eurythoe complanata* (Pallas, 1766).

#### Eurythoe laevisetis

Fauvel, 1914

http://species-id.net/wiki/Eurythoe_laevisetis

Fig. 2A–F

Eurythoe laevisetis Fauvel, 1914: 116, pl VIII fig. 28-30, 33-37. Type locality: São Tomé Island, Gulf of Guinea.

##### Material examined.

*Eurythoe* cf. *complanata*: Gozo Harbour (Malta), 35°50'N, 14°35'E (Mar. 2011): BOS-Amp3 (2 specimens), BOS-Amp4 (9 specimens).

*Eurythoe complanata*: Isabel II Island (Chafarinas Islands, Spain), 35°11'N, 2°26'W (Sep. 1992): MNCN 16.01/3340 (1 specimen); (Jul. 1993) MNCN 16.01/33394 (1 specimen).

##### Diagnosis and description.

**Figure 2.**
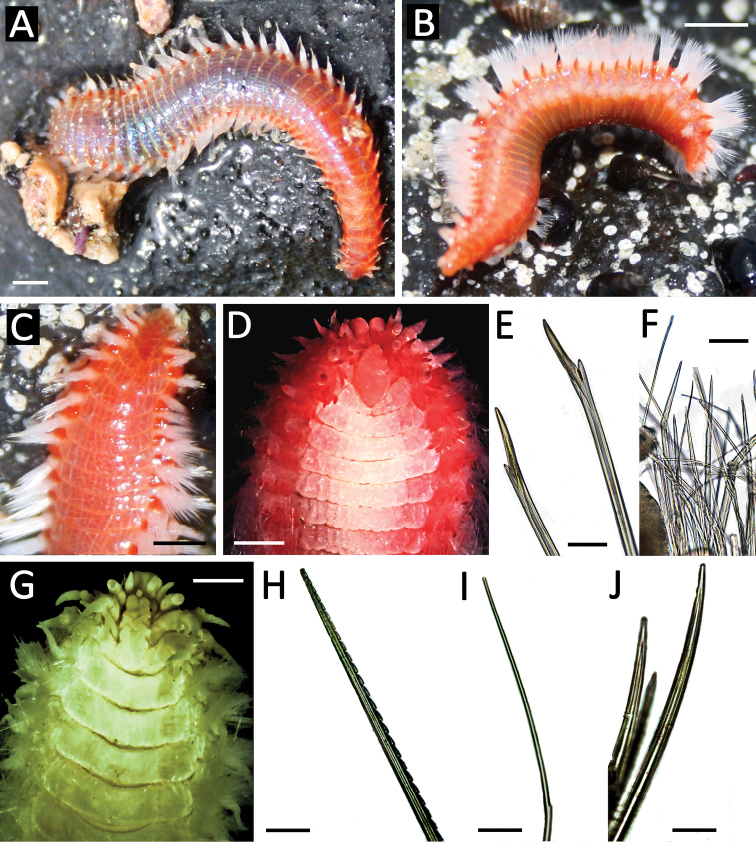
*Eurythoe laevisetis* from Malta. **A** live specimen, general view **B** live specimen, lateral view **C** live specimen anterior end, dorsal view **D** detailed view of anterior end, dorsal view **E** neurochaetae **F** spurred capillary notochaetae. *Eurythoe complanata* from Israel **G** detailed anterior end, dorsal view **H** harpoon notochaeta **I** notopodial spurred capillar notochaeta **J** notoacicular spines.

Body depressed elongated, rectangular in cross section. Specimens from Malta ranged in length from 14 to 52 mm with a mean of 39 mm (N=11, SD=12.09). Live specimens have a uniform orange-pinkish colour (Fig. 2A–C), on which the gills and a bright red caruncle stand out, and white chaeta fascicles forming two longitudinal bands along the body (Fig. 2A, B). Prostomium rounded with 2 pairs of inconspicuous eyes arranged in a square and three antennae, two lateral ones in an anterior position and one slightly behind the others. The anterior end has a bilobed prebuccal lobe where are inserted a pair of cirriform palps (Fig. 2D). The caruncle is elongated and extends until the third chaetiger (Fig. 2C, D). Each segment is provided with a pair of arborescent gills that are present from the second chaetiger to the posterior region (Fig. 2C, D). Biramous parapodia with digitiform dorsal and ventral cirri, similar in size. Notochaetae of two types: very fine with a small spur that continues in a capillary-like thorn; and thicker with a marked spur (spurred capillary notochaeta) (Fig. 2F). The neurochaetae are spur-type and thick, slightly denticulate on juveniles (Fig. 2E).

##### Remarks.

Several Maltese specimens present evidence of regeneration of the anterior and posterior end. All preserved specimens have whitish colour and lack the characteristic harpoon notochaetae. The two pairs of eyes are extremely inconspicuous, the anteriormost being similar in size to the posterior one. Specimens from Malta and Chafarinas Islands were morphologically identical to the Atlantic *Eurythoe laevisetis* from the Canary Islands and Cape Verde and *Eurythoe laevisetis* from São Tomé Island.

#### Eurythoe complanata

(Pallas, 1766)

http://species-id.net/wiki/Eurythoe_complanata

Fig. 2G–J

Aphrodita complanata Pallas, 1766: 109, pl. 8, fig. 19-26. Type locality: Antigua Island, Caribbean Sea.
Eurythoe brasiliensis Hansen, 1882: 4, fig. 5-9.
Lycaretus neocephalicus Kinberg, 1867: 55-56.
Eurythoe kamechameha Kinberg, 1857: 14; 1910: 36, pl. 12, fig. 13.
Eurythoe pacifica Kinberg, 1857: 14; 1910: 36, pl. 12, fig. 11.
Eurythoe indica Kinberg, 1867: 90.
Eurythoe alboseta Kinberg, 1857: 90.
Eurythoe ehlersi Kinberg, 1867: 90.
Eurythoe havaiva Kinberg, 1867: 90.
Eurythoe corallina Kinberg, 1857: 14; 1910: 36, pl. 12, fig. 12.
Eurythoe alcyonaria Gravier, 1902: 83, fig. 38, b-m.

##### Material examined.

*Eurythoe complanata*: Isabel II Island (Chafarinas Islands, Spain), 35°11'N, 2°26'W (Sep. 1992): MNCN 16.01/3337 (2 specimens), MNCN 16.01/3338 (2 specimens), MNCN 16.01/3340 (1 specimen); Congreso Island (Chafarinas Islands, Spain), 35°11'N, 2°26'W (Jul. 1993): MNCN 16.01/3336 (1 specimen); Isabel II Island (Chafarinas Islands, Spain), 35°11'N, 2°26'W: MNCN 16.01/33394 (2 specimens). Atlit (Israel), 32°41'N, 34°56'E (1937): BMNH 1937.4.7.1-5 (7 specimens).

##### Diagnosis and description.

Israeli specimens ranged from 20 to 45 mm in length with a mean of 31 mm (N=7, SD=9.77). Prostomium rounded with 2 pairs of eyes arranged in a square, the first being larger (Fig. 2G), and with three antennae, lateral ones in an anterior position and the single one slightly posterior. Anterior end with a bilobed prebuccal lobe, carrying a pair of cirriform palps. The caruncle is elongated and extends until the third chaetiger (Fig. 2G). Each segment is provided with a pair of arborescent branchiae that are present from the second chaetiger to the posterior end. Biramous parapodia with dorsal and ventral cirri digitiform, similar in size. Notochaetae of three types: harpoon-like (Fig. 2H); spurred capillaries with small spurs (Fig. 2I) and thicker smooth notochaetal spines (Fig. 2J). Notoacicula are very small, hastate, limited in number and always form an arc immediately in front of the dorsal cirrus. Neurochaetae are bifurcate, with prongs of different lengths.

##### Remarks.

One specimen regenerating the posterior end. Pairs of eyes inconspicuous in some specimens, but always with the anterior pair larger than posterior pair. Specimens from Chafarinas Islands had a mean size of 37 mm (N= 8, SD = 7.24). All preserved specimens had a brownish colour.

### Key to genera of Amphinomidae and species of *Eurythoe* and *Linopherus* of the Mediterranean Sea (modified from Borda et al. 2012)

**Table:** 

1	Caruncle absent	*Hipponoa*
–	Caruncle present, variably developed	2
2	Oval body	3
–	Elongated body; subcylindrical or quadrangular cross section	4
3	Dorsal accessory (branchial) cirri plus dorsal cirri on anteriormost abranchiate chaetigers; in branchiate chaetigers, one dorsal cirri per notopodium; bipinnate branchiae	*Chloeia*
–	Dorsal accessory (branchial) cirri plus dorsal cirri on all chaetigers; palmate branchiae	*Notopygos*
4	First chaetiger dorsally continuous, complete	5
–	First chaetiger dorsally discontinuous, not complete	7
5	Hooks present in the first chaetiger; caruncle round	*Paramphinome*
–	Hooks not present in the first chaetiger	6
6	Branchiae limited to anterior segments	*Linopherus* 10
–	Branchiae on all segments after the chaetiger 2 or 3	*Amphinome*
7	Caruncle large and conspicuous, extending beyond one chaetiger posteriorly	8
–	Caruncle small and inconspicuous, not extending beyond one chaetiger posteriorly	*Cryptonome*
8	Caruncle without a median lobe, with folds obliquely arranged	*Hermodice*
–	Caruncle with a smooth median lobe	9
9	Caruncle not sinusoidal	*Eurythoe* 11
–	Caruncle sinusoidal	*Pareurythoe*
10	First branchiae present on chaetiger 3	*Linopherus canariensis*
–	First branchiae present on chaetiger 4	*Linopherus acarunculatus*
11	Three types of notochaetae present: spurred capillary, notoacicular spine and harpoon	*Eurythoe complanata*
–	Two types of notochaetae present: spurred capillary and notochaetal spine; harpoon absen	*Eurythoe laevisetis*

## Discussion

Members of the family Amphinomidae have a number of characteristics that gives the group high invasive potential. They show high biological plasticity and reproductive habits that include both sexual and asexual reproduction; possess a great capacity of regeneration and a large dispersal capability due to their long-term rostraria larvae (Kudenov 1995, Cosentino and Giacobbe 2011). Four amphinomid species are currently considered to be established in the Mediterranean Sea: *Eurythoe laevisetis*, *Eurythoe complanata*, *Linopherus canariensis* and *Linopherus acarunculatus* (Table 1). *Notopygos crinita* is presumably no longer present in the Mediterranean Sea, having been a case of accidental introduction that failed to establish (Zenetos et al. 2010, 2012, Occhipinti-Ambrogi et al. 2011). However, the recently introduced *Linopherus canariensis* has displayed a highly invasive capacity and great potential for colonization, which are particularly favoured in stressed and degraded habitats where populations reach densities over 42 individuals/m^2^ (Cosentino and Giacobbe 2011).

The use of the term ‘morphospecies’ for referring to *Eurythoe complanata* has been proposed as an alternative to overcome the identification difficulties associated with this species complex, which includes two cryptic species along with *Eurythoe laevisetis*. Here, we have an example of two species that are genetically distinct but morphologically identical under the same ‘morph’, named as *Eurythoe complanata*. So, the *Eurythoe complanata* complex erected by Barroso et al. (2010) is actually formed by two morphospecies, *Eurythoe complanata* and *Eurythoe laevisetis*. The former includes two cryptic species which occur natively, one in the eastern Pacific and one in the Atlantic.

The *Eurythoe complanata* complex represents one more case of species group that is likely to be introduced in the Mediterranean, but which has been underestimated and misidentified. Re-examination of specimens from Malta, Chafarinas Islands and Israel demonstrates the existence of two morphospecies belonging to the *Eurythoe complanata* complex in the Mediterranean Sea: *Eurythoe laevisetis* in the western and central Mediterranean and *Eurythoe complanata* in the western and Levantine basins. Moreover, the Israeli *Eurythoe complanata* is not a recently introduced species, but one that had been present since, at least 1937. All examined specimens from Malta and two from Chafarinas Islands belong to the species *Eurythoe laevisetis*, characterized by the absence of the harpoon notochaetae. According to Barroso et al. (2010), the ‘Atlantic-island-restricted species’, differentiated by DNA sequences and morphology from *Eurythoe complanata* is, actually, *Eurythoe laevisetis*. This species was erroneously considered the junior synonym of *Eurythoe complanata* by several authors (e.g. Fauvel 1947, Ebbs 1966). According to Fauvel (1914), the main diagnostic feature distinguishing *Eurythoe laevisetis* from the related *Eurythoe complanata* is its lack of harpoon notochaetae (Barroso et al. 2010). After the examination of the *Eurythoe laevisetis* specimens (without harpoon notochaetae) from different localities (Malta, Chafarinas Islands, Canary Islands, Cape Verde and São Tomé Island), we observed that both anteriormost and posterior pairs of prostomial eyes were similar in size in all studied specimens, being always very inconspicuous. By contrast, all examined specimens belonging to *Eurythoe complanata* exhibited, besides the characteristic harpoon chaetae, anterior eyes larger than posterior ones.

On the other hand, all examined specimens from Israel and nine from Chafarinas Islands were morphologically identical to *Eurythoe complanata* from the Atlantic and Pacific *sensu* Barroso et al. (2010), including the characteristic harpoon notochaetae, length of caruncle, prostomial appendages, branchial distribution pattern and other types of notopodial and neuropodial chaetae. These specimens differ from *Eurythoe laevisetis* by the presence of the harpoon notochaetae and size differences between the two pairs of eyes, with the anterior pair always larger than the posterior ones.

Kinberg (1857) first described the genus *Eurythoe* in the Mediterranean Sea based on *Eurythoe syriaca* from the Syrian coasts and *Eurythoe hedenborgi* from Dr. Hedenborg’s collection. Later, Monro (1937) reported *Eurythoe complanata* for the first time from the Mediterranean, considering *Eurythoe syriaca* as its junior synonym. Nevertheless, Hartman (1948) when reviewing the species described by Kinberg considered *Eurythoe syriaca* as a valid species. In the same review, as well as in her later world catalogue Hartman (1959) regarded *Eurythoe hedenborgi* as a questionable species, even though no justification was provided. More recently, Çinar (2008) described *Eurythoe turcica* from the Levantine coast of Turkey and differentiated this species from the related Indo-Pacific *Eurythoe parvecarunculata* Horst, 1912. Nevertheless, Borda et al. (2012) transferred these latter two species to the genus *Cryptonome* based on a phylogenetic analysis. Therefore, based upon a comprehensive review of the literature descriptions we propose that currently only two species can be validly assigned to the genus *Eurythoe* in the Mediterranean Sea, *Eurythoe complanata* and *Eurythoe laevisetis*.

The origins, plausible pathways and introduction vectors of these related amphinomids into the Mediterranean may be discerned by focusing on populations of the central (*Eurythoe laevisetis*), western (*Eurythoe laevisetis* and *Eurythoe complanata*) and Levantine (*Eurythoe complanata*) regions. For example, Maltese and Chafarinas populations of *Eurythoe laevisetis* may have originated from Atlantic islands through the Gibraltar Strait. Such a scenario is wholly consistent with arrivals of other Atlantic species of marine invertebrates into the Mediterranean such as the gastropod *Marginella glabella* (Linnaeus, 1758), which is presently colonizing the coasts of Málaga (SE Spain, western Mediterranean) from the Canary Islands and West Africa (Luque et al. 2012). The Gibraltar Strait was also suggested to be the main pathway of introduction for other polychaetes such as the invasive sabellid *Branchiomma bairdi* (McIntosh, 1885), which is associated with *Eurythoe laevisetis* in Maltese Islands (Arias et al. 2013a) and for other conspicuous amphinomids, such as *Hermodice carunculata*. The Mediterranean populations of the latter also seem to have descended from Atlantic ones (Ahrens et al. 2013) as well as *Linopherus canariensis* populations from the Italian coasts (Cosentino and Giacobbe 2011). Two different plausible hypotheses concerning *Eurythoe complanata* populations must be considered in relation to their present geographical distributions. For example, Israeli populations could be Lessepsian migrants due to their proximity to the Suez Canal. On the other hand, *Eurythoe complanata* from the Chafarinas islands and also localized in the Strait of Gibraltar, could be Atlantic migrants from the Canaries or other Atlantic archipelagos. However, multiple routes and times of introduction for all studied populations (Chafarinas, Malta and Israel) seem tenable and cannot be excluded. Further research mainly using molecular markers of Maltese and Israeli populations, as well as Red Sea and Canary Island ones, is needed to give more information concerning their origins and dispersion in the Mediterranean Sea. Finally, it is essential to emphasize that the great dispersive capacity of *Eurythoe complanata* (Barroso et al. 2010) is likely due to the inferred high longevity of its planktotrophic rostraria larvae (Bhaud 1972); additionally, the combination of asexual and sexual reproduction (Kudenov 1974) may promote the invasive potential of this species. Therefore, a detailed monitoring of the dynamics of Maltese and Israeli populations, as well as setting up a current distribution map should be undertaken in order to establish and understand the evolution of *Eurythoe complanata* complex across the Mediterranean Sea.

## Supplementary Material

XML Treatment for Eurythoe laevisetis

XML Treatment for Eurythoe complanata
